# *In vivo* screening and discovery of novel candidate thalidomide analogs in the zebrafish embryo and chicken embryo model systems

**DOI:** 10.18632/oncotarget.8909

**Published:** 2016-04-22

**Authors:** Shaunna L. Beedie, Holly M. Rore, Shelby Barnett, Cindy H. Chau, Weiming Luo, Nigel H. Greig, William D. Figg, Neil Vargesson

**Affiliations:** ^1^ School of Medicine, Medical Sciences and Nutrition, Institute of Medical Sciences, University of Aberdeen, Foresterhill, Aberdeen, UK; ^2^ Molecular Pharmacology Section, Genitourinary Malignancies Branch, Center for Cancer Research, NCI, NIH, Bethesda, Maryland, USA; ^3^ Drug Design and Development Section, Translational Gerontology Branch, Intramural Research Program, National Institute on Aging, National Institutes of Health, Baltimore, Maryland, USA; ^4^ Current address: Centre for Applied Pharmacokinetic Research, Manchester Pharmacy School, University of Manchester, Manchester, UK

**Keywords:** angiogenesis, inflammation, thalidomide, cancer, teratogenesis

## Abstract

Thalidomide, a drug known for its teratogenic side-effects, is used successfully to treat a variety of clinical conditions including leprosy and multiple myeloma. Intense efforts are underway to synthesize and identify safer, clinically relevant analogs. Here, we conduct a preliminary *in vivo* screen of a library of new thalidomide analogs to determine which agents demonstrate activity, and describe a cohort of compounds with anti-angiogenic properties, anti-inflammatory properties and some compounds which exhibited both. The combination of the *in vivo* zebrafish and chicken embryo model systems allows for the accelerated discovery of new, potential therapies for cancerous and inflammatory conditions.

## INTRODUCTION

Thalidomide was first marketed in Germany in 1957 as a non-barbiturate, non-addictive, non-toxic sedative which was also used to treat morning sickness in pregnant women [1–4]. It was withdrawn from the world market in late 1961 after it was found to be a potent teratogen [2–4] having caused birth defects in over 10,000 children. These children showed similar anatomical limb reduction malformations including phocomelia, an absence or reduction of the long bones in the forelimb, or amelia, a complete absence of the forelimb [2, 4, 5]. Other commonly seen phenotypic malformations were also documented including eye, ear, heart, gastrointestinal and kidney defects [2, 6–8]. The thalidomide disaster emphasized the importance of thorough drug screening and the potential for drug species specificity, as rodents were not susceptible to thalidomide. As a direct result new candidate drugs are now tested in at least two animal species (one of which must be non-rodent) before clinical testing [9].

Thalidomide possesses anti-angiogenic actions in the rabbit corneal micropocket assay [10], the rat aortic ring assay (in the presence of human liver microsomes), and in *in vitro* cultures of human umbilical vein endothelial cells (HUVECs), [6, 11–15]. This action has been linked to the damage to embryos following thalidomide exposure [3, 6, 8, 10]. Thalidomide also possesses immunomodulatory characteristics [16, 17]. The immunological basis for the clinical efficacy of thalidomide lies in its ability to inhibit the synthesis of tumor necrosis factor alpha (TNF-α), a major inflammatory cytokine [17, 18]. Thalidomide's combined anti-angiogenic and anti-inflammatory properties likely lead to its anti-cancer effects and efficacy in the treatment of multiple myeloma [19, 20] as well as documented activity in other cancers [21].

Given the efficacy of thalidomide analogs and expanding clinical use, developing safer thalidomide analogs with improved activity and less toxicity is a continuing research effort. The novel analogs used in the current study were designed around the tricyclic ring structure of thalidomide. A library of 81 structural analogs were chemically synthesized and screened in the zebrafish embryo and chicken embryo model systems. Our study utilizes two established transgenic zebrafish reporter lines, *fli1*:EGFP and Tg(*mpo*::EGFP)^114^, to identify potential anti-angiogenic and anti-inflammatory compounds, respectively [12, 22-25]. Here we describe the 13 lead compounds that exhibit either anti-angiogenic, anti-inflammatory, or both, properties. The multiple zebrafish assays, used in conjunction with the *in ovo* chicken embryo assay, also help to provide a potential teratogenic profile for each lead compound.

## RESULTS

### Anti-angiogenic thalidomide analogs disrupt vessel formation in fli1:EGFP zebrafish embryos

**Figure 1:**
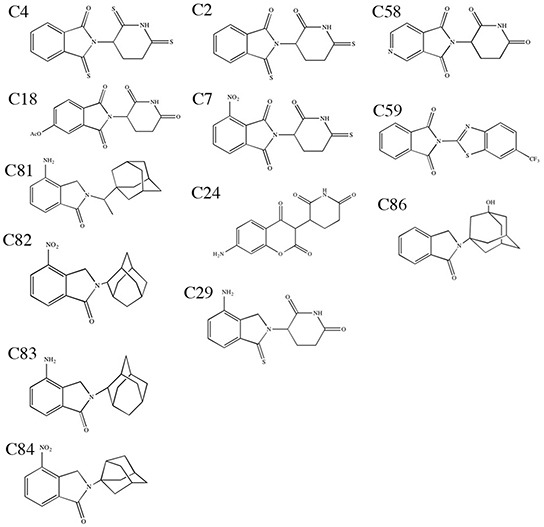
Structures of compounds of interest Structures of lead thalidomide analogs of interest identified in our models.

**Figure 2:**
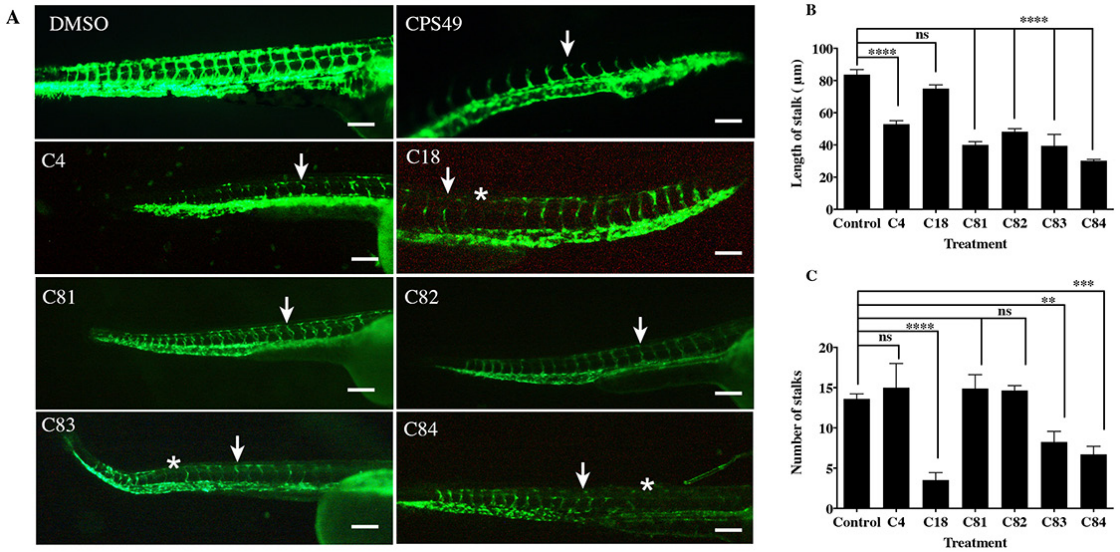
The effect of novel thalidomide analogs on vessel length *fli1*:EGFP zebrafish were incubated with vehicle or compounds for 24 hours, at which point vessel outgrowth and number were measured. **A.** Images of zebrafish exposed to the vehicle or test compounds. Zebrafish with the vehicle (0.1% DMSO) show normal patterning of the ISVs, zebrafish exposed to CPS49 show reduction in vessel outgrowth (white arrow), loss of vascular connectivity and a decrease in the number of forming blood vessels. Treatment with C4 (1.5 μg/mL), C18 (50 μg/mL), C81 (50 μg/mL), C82 (5 μg/mL), C83 (20 μg/mL) and C84 (50 μg/mL) show reduction in vessel out growth (white arrow) and in some cases loss of vessel (white asterisks). **B.** Stalk length & **C.** stalk number are reduced. Compounds are shown at the lowest concentration producing an effect. The data represent the mean + the standard error of the mean.

To determine if our novel thalidomide analogs (Figure 1) had any effect on *in vivo* angiogenesis, we used *fli1*:EGFP embryos which express green fluorescent protein (GFP) on endothelial cells throughout development [22]. Embryos were allowed to develop for 24 hours, then exposed to either vehicle or a compound of interest at the concentration range of 1 to 200 μg/mL. During this time the vasculature is rapidly expanding and developing new, naive intersegmental vessels (ISVs). The embryos were imaged after 24 hours of incubation with the drug (Figure 2A). Compounds C4, C18, C81, C82, C83 and C84 exhibited anti-angiogenic activity, as indicated by loss of ISVs or inhibition of ISV outgrowth in *fli1*:EGFP zebrafish embryos (Figure 2B–2C). We also observed that the potency of each compound varied. Table 1 shows the lowest effective concentration of each compound exhibiting a response. For example, C4 was anti-angiogenic at 1.5 μg/mL (4.9 μM), as determined by inhibition of vessel outgrowth and number. In contrast C81 only showed inhibitory activity at 50 μg/mL (161.07 μM).

**Table 1:** Lowest effective concentration determined for the 11 lead thalidomide analogs screened in each assay

	Compound	Assay			
		*fli1*:EGFP	TG(MPO::EGFP)^114^	WT Zebrafish	Chicken
Activity		(μg/mL)	(μg/mL)	(μg/mL)	(μg/mL)
Anti-angiogenic	4	1.5	10	10	100
	81	50	50	10	100
	83	20	20	10	100
	84	50	10	10	100
Anti-inflammatory	2	10	10	10	100
	7	10	10	10	100
	24	50	50	50	100
	29	10	5	nd	100
	58	10	5	5	100
	59	10	100	100	50
	86	10	100	nd	100
Both	18	50	50	50	100
	82	5	5	5	100

Compounds were screened across a range of concentrations (1-200 μg/mL) to determine the lowest effective concentration. Where no activity was seen the concentration shown is the lowest screened.

### Anti-inflammatory thalidomide analogs reduce neutrophil migration in response to injury in the Tg(MPO::EGFP)^114^ zebrafish

**Figure 3:**
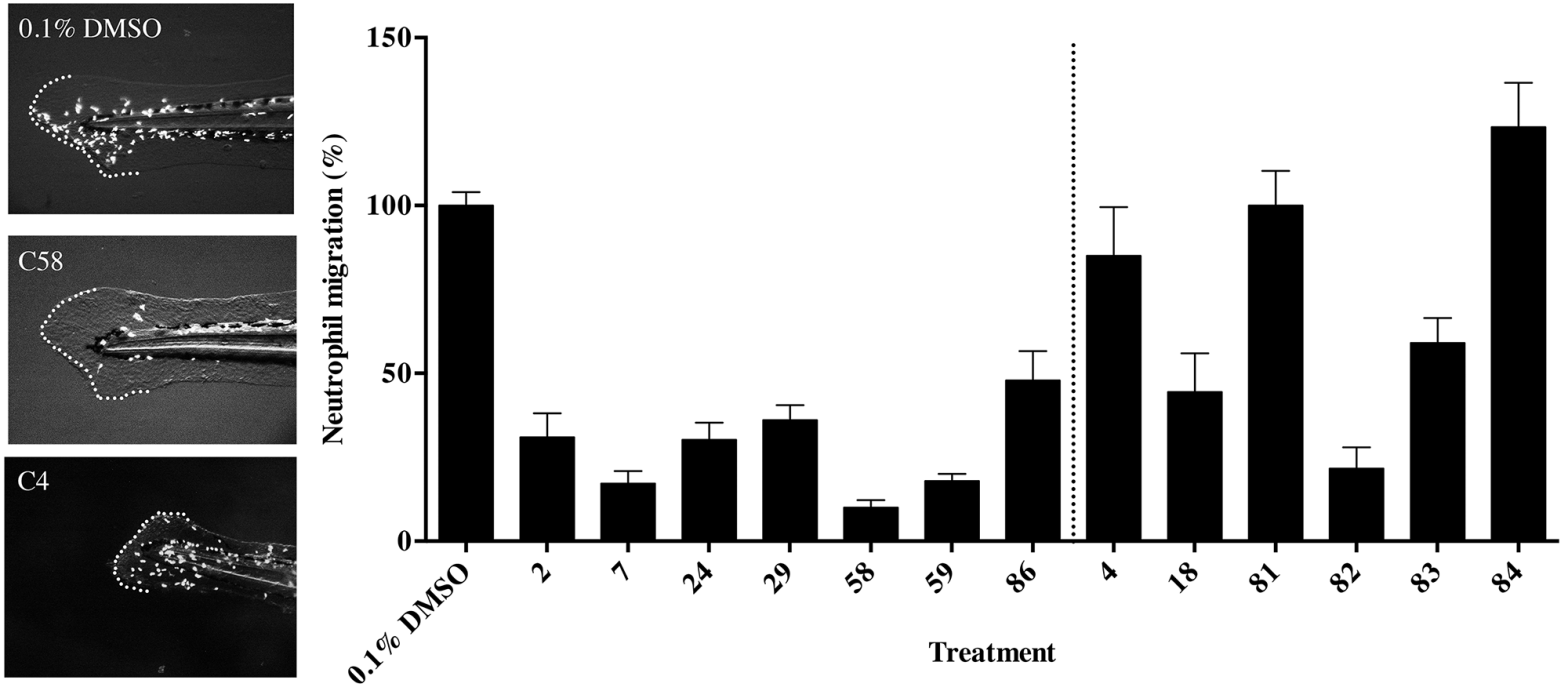
The effect of thalidomide analogs on the inflammatory response to wound healing **A.** Zebrafish (72 hpf) embryos transgenic for a fluorescently tagged neutrophil marker Tg(MPO::EGFP)^114^ were fin clipped to induce an inflammatory response and exposed to vehicle or a test compound. Broken line indicates the tail fin with cut and area of the wound site. **B.** The percentage of neutrophils within the wound site was quantified and shows the extent of the inflammatory response to injury. The data represent the mean + the standard error of the mean.

To assess the immunomodulatory effects of the thalidomide analogs across the concentration range used in the previous assay, the transgenic zebrafish line Tg(MPO::EGFP)^114^ was used. These embryos express GFP tagged myeloperoxidase, marking neutrophils during the inflammatory response, for example following injury [23, 24]. The inflammatory response in zebrafish embryos is active from 3 days post fertilization [24] and can clearly be demonstrated following removal of the dorsal third of the tail fin. This allowed us to subsequently assess the anti-inflammatory activity of each compound by immersing embryos in the test drug or vehicle. The embryos were examined for migration of neutrophils to the wound site. In comparison to control embryos (Figure 3A) compounds with anti-inflammatory properties had reduction of at least 50% of neutrophils recruited to the wound site (Figure 3). Compounds C2, C7, C24, C29, C58, C59 and C86 reduced neutrophil migration. We also screened the previously identified six anti-angiogenic compounds of interest in the Tg(MPO::EGFP)^114^ line. C18 and C82 were seen to significantly reduce neutrophil migration, and thus were classified as having both anti-angiogenic and anti-inflammatory properties in these assays (Figure 3B).

### All anti-angiogenic and some anti-inflammatory compounds are teratogenic in the developing chicken and zebrafish model systems

**Figure 4:**
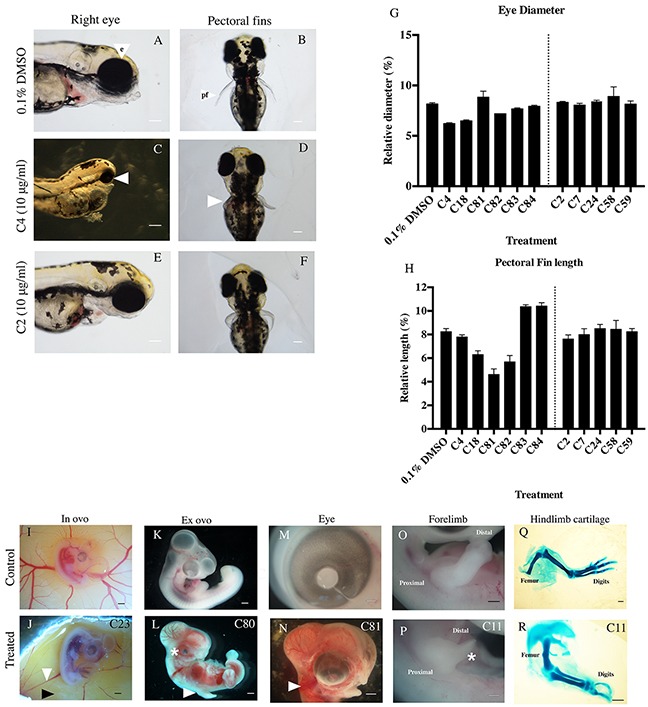
Defects seen in zebrafish and chicken embryos following thalidomide analog treatment **A.** Embryos with exposure to a vehicle control show normal development of the eye (e), and **B.** pectoral fins (pf). Example of an anti-angiogenic compound (C4, Figure 1) causing **C.** microopthalmia (white arrow; seen in list the compounds) and **D.** malformation in fin development (white arrow). Exposure to an anti-inflammatory compound (C2, Figure 1) resulting in **E.** normal eye development and **F.** fin development. **G.** Eye diameter and **H.** fin length were quantified and show anti-angiogenic compounds causing reductions in eye diameter (C4, C18, C82, C83, C84) and pectoral fin length (C18, C81, C82). Compounds of interest were screened in chicken embryos at HH stage 17-18 (Day 2.5 in embryonic development). **I.** Untreated, control images of an embryo *in ovo* with normal vascular patterns at HH stage 23 (Day 3.5); **K.** *ex ovo*; **M.** eye **O.** forelimb and **Q.** hindlimb showing cartilage patterning (at day 9 of development). Typical examples of compound treated embryos: **J.** anomaly in vasculature (white arrow) and necrosis (black arrow) of the YSM in a chicken embryo following treatment with C23 (Supplementary Table S1). **L.** embryo exhibiting microopthalmia (asterix), limb reduction (white arrow) and hemorrhaging throughout the body following treatment with C80 and (N) C81 (Figure 1) treated embryo with growth reduction and hemorrhaging throughout head (white arrow). **P.** C11 treated limb with reduced elements (Asterisks represents a limb reduction defect). **R.** Missing digits and reduced length of limb cartilage elements (Treated with C11, Supplementary Table S1). Scale bar represents 1000 μm.

Compounds of interest were screened in chicken and/or zebrafish embryos (Figure 4), to determine if these compounds are teratogenic. The chicken embryo is well established as a model to determine the action of compounds upon development [6, 12, 23, 25–27]. All the identified anti-angiogenic compounds (Structures in Supplementary Table S1) caused defects to the embryos. Teratogenicity in the chicken embryo included microphthalmia (compound C81), reduced body size compared to control embryos (compound C18), limb and digit defects (compound C82), and hemorrhaging (compounds C4, C81, C83, C84, C24) (Table 2, Figure 4). Chicken embryos treated with these compounds often had abnormal blood vessels and necrotic-like regions in the surrounding yolk sac membrane (compounds C4, C18, C83, C24).

**Table 2:** Damage seen in HH stage 17-18 chicken embryos when exposed to compounds of interest at 100 μg/mL

Compound	Damage observed	Survival (n)
C4	Necrotic YSM, hemorrhaging	1/10
C18	Necrotic YSM, stunted growth	4/6
C81	Hemorrhaging, microopthalmia,	3/12
C82	Limb reduction defect	3/8
C83	Necrotic YSM, hemorrhaging	2/4
C84	Hemorrhaging	2/4
C24	Necrotic YSM, hemorrhaging	2/4
C29	Twisted spines, hemorrhaging	7/11

To determine the effects of the lead candidate compounds on embryonic development, compounds were tested in the developing zebrafish model during a stage of rapid organogenesis (24 hpf) [23]. Anti-angiogenic compounds caused developmental issues in the zebrafish embryo, including reducing eye diameter (Figure 4C, Figure 4G), pectoral fin outgrowth malformations (Figure 4D, Figure 4H) and twisted spines (Figure 4C).

Of the lead anti-inflammatory compounds, compounds C24 and C29 exhibited developmental defects in chicken embryos causing hemorrhaging and necrotic-like damage in the YSM. Compounds C2, C7, C58, C59 and C86 had no effect on chicken embryo development.

## DISCUSSION

Thalidomide and its analogs are currently under investigation for the treatment of a range of auto-immune and inflammatory conditions, as well as certain cancers [28]. The continued use of thalidomide has emphasized the need for an alternative form of the drug with increased clinical potency and bioactivity, but with reduced side effects. This need is further magnified by a recent resurgence of children born in Brazil with thalidomide-induced defects, which has increased efforts to design and synthesize a non-teratogenic thalidomide analog [29–32].

We have assessed the *in vivo* actions of novel thalidomide analogs in zebrafish and chicken embryo model systems. Utilizing these assays we have screened 81 compounds and shown that a subset of analogs displayed desirable actions at low concentrations *in vivo*. We have selected 13 lead candidate compounds from these cohorts based on their activity profiles.

Compounds C4, C18, C81, C82, C83 and C84 were found to decrease vessel length or the number of sprouting vessels at relatively low concentrations (1.5 μg/mL - 100 μg/mL), similar to concentration ranges used in other screening studies [23, 25, 33]. Some compounds with anti-angiogenic actions were found to induce death and defects within the treated embryos, reflecting the importance of vascular development for embryogenesis. This complements other studies that suggest a primary cause of thalidomide-induced birth defects may be incorrect patterning of the vasculature in affected tissues [6, 12].

We identified several analogs with potent activity against the inflammatory response *in vivo* at low concentrations compared to previous studies of other thalidomide analogs [23]. Of these compounds, C2, C7, C58, C59 and C86 were found to be anti-inflammatory without anti-angiogenic activities and were non-teratogenic in these assays, and thus may be acting independently of the mechanism that induces defects in the developing embryo. Importantly, we cannot rule out that at higher concentrations, or in other animal models, they could be teratogenic. C58 was the most effective analog at inhibiting the inflammatory response, decreasing the migration of neutrophils to 10% of the control (Figure 3). Interestingly, the structure of C58 (5-Aza-thalidomide) is very similar to pomalidomide, a derivative of thalidomide with potent immunomodulatory actions [34, 35]. However, unlike pomalidomide, C58 includes a pyridine ring. Whether this analog shares a similar mechanism of action to pomalidomide remains to be determined. Of additional note, C2 (2,3-dihydro-2-(2-oxo-6-thioxo-3-piperidinyl)-3-thioxo-1H-isoindol-1-one) also possessed significant anti-inflammatory action. This agent, also known as 3,6′-dithiothalidomide, has been shown to potently lower lipopolysaccharide (LPS)-induced elevations in TNF-α in cultured RAW 264.7 cells as well as in the plasma and brain of rats challenged with systemic LPS [33, 36]. The compound also dramatically mitigates neuroinflammation and improves outcome measures in animal models of traumatic brain injury [37], stroke [38], Alzheimer's disease [33, 39, 40] and aneurysm [41]. Together, these prior studies cross-validate the anti-inflammatory actions evident within the present study. While most anti-inflammatory compounds did not induce embryonic defects, C24 and C29 caused some effects in the developing chicken embryo including twisted spinal cords, necrotic-like damage in the YSM and high death rates. Thalidomide inhibits TNF-α expression which is vital for the induction of an inflammatory response [42]. Given that TNF-α can be protective to rodent embryos exposed to teratogenic insults [43] it may also be that a reduction of TNF-α somehow impedes the development of the chicken and zebrafish embryos. Further studies elucidating this developmental mechanism would aid in the use of chicken and zebrafish embryos for the screening of developmental toxicity and embryotoxic potency of compounds (e.g. other TNF-α inhibitors) during early drug development. Reports on the risk of birth defects after embryonic exposure to TNF-α inhibitors are contradictory, with some indicating they may be teratogenic [44] and other studies reporting their safety [45, 46]. It is important to point out that thalidomide exerts its anti-inflammatory effects through multiple pathways including TNF-α and COX-2 inhibition. The fact that our current study identified some anti-inflammatory thalidomide analogs that produced mild defects (compared with thalidomide embryopathy in humans) suggests that the effects of thalidomide and its analogs on the embryos may involve multiple targets. Using our screening models, we can identify those that are potently anti-inflammatory analogs but are non-teratogenic. Given the species specificity of thalidomide and that animal studies do not always correlate to human responses, extrapolation of this data should be carried out with caution.

Several compounds from our library, such as C18 and C82, exhibited both anti-inflammatory and anti-angiogenic properties in our assays (structures found in Supplementary Table 3). Tumors require a blood supply to survive [47] and the tumor microenvironment includes a high level of cytokine activity (including COX-2, TNF-α and interleukins). Therefore, compounds inhibiting both vessel formation and the inflammatory response may be beneficial for anti-cancer therapy [48, 49].

The effects of novel thalidomide analogs in this study provide primary data as evidence for their clinical potential. Studies are underway in our laboratory for further testing in *in vitro* cellular and *in vivo* animal models of various disease states as well as to delineate the molecular mechanisms of the lead compounds. In particular, if the compounds act by binding to cereblon, a recently established molecular target of thalidomide, lenalidomide and pomalidomide [50, 51]. Given that these compounds could be divided into distinct cohorts based on their activities we hypothesized there may be some correlation between the structural modifications and their activity in these assays. To assess this across the library of compounds we are conducting structure-activity relationship studies. Due to the range of teratogenicity seen in the developing chicken model system with some of these thalidomide-related compounds, it will be interesting to assess whether or not the compounds have a direct effect on cells or if the anti-cancer action is purely mediated by the inhibition of angiogenesis. While it has been shown in previous studies that thalidomide exhibits multiple effects [52], the compounds screened here will require additional characterization to establish if they also exert sedative or neurotoxic effects in addition to their anti-inflammatory or anti-angiogenic properties. The iterative process of continued synthesis and screening of thalidomide analogs increases the chances of finding a form of the drug that acts with tissue specificity, increased potency and reduced unwanted effects.

## MATERIALS AND METHODS

### Zebrafish embryology

Zebrafish embryos were treated with analogs as previously described [23]. Briefly, *fli1*:EGFP embryos were collected and embryos were allowed to develop for 24 hours. Embryos were dechorionated manually and exposed to test compounds or vehicle control (0.1% DMSO) for a further 24 hours. Embryos were anesthetized with 0.05% MS222 (Tricaine) (Sigma Aldrich) and live visualization of blood vessels was carried out.

Tg(MPO::EGFP)^114^ embryos were tail fin clipped as previously detailed [23, 24] and incubated with test compounds or vehicle control at 72 hpf. Fish were imaged at 24 hours and the number of migratory neutrophils present at the wound site were counted. Compounds inducing an at least 50% reduction of neutrophils to the wound site were considered to have anti-inflammatory properties in this system. All larvae were assessed for viability and morphological integrity. A minimum of 10 embryos were used per concentration per drug. Error bars represent standard error of the mean.

### Chicken embryology

Fertilized white leghorn chicken embryos were incubated at 38°C and staged according to the Hamburger and Hamilton (HH) stages of development [53]. Embryos were tested at HH stage 17-18 (day 2.5). Following membrane removal test compounds or vehicle control solutions were applied over the embryo. The eggs were sealed and the development of the embryos was monitored up to HH stage 30 (E9). A minimum of 3 embryos were used per concentration, per drug.

All embryo work was fully licensed and carried out with ethical review permissions and following all regulatory guidelines.

### Thalidomide analogs

A broad series of novel thalidomide-based compounds were chemically synthesized (WL, NHG and WDF, NIH), dissolved in DMSO, stored in stock concentrations between 10-100 mg/mL, and used at a final working DMSO concentration of 0.1%. The chemical structures of lead compounds of interest were confirmed by chemical characterization (purity >99.5%). Compounds were screened across a range of concentrations from 1-200 μg/mL to determine the lowest effective concentration.

### Imaging and analysis

Imaging was performed using a Nikon MZ1500 fluorescent stereomicroscope with a Nikon DS-5 digital camera and analyzed using Adobe photoshop and Image J. Analysis was conducted using Prism 6.0 (GraphPad Software, La Jolla, CA) and statistical significance was assessed using two-tailed Student's *t* tests or ANOVA analyses. Error bars represent standard error of the mean.

## SUPPLEMENTARY TABLES


