# Supplementary material for: In vivo screening and discovery of novel candidate thalidomide analogs in the zebrafish embryo and chicken embryo model systems
Source: Oncotarget. 2016 Apr 22;7(22):33237–45. doi: 10.18632/oncotarget.8909 (PMC5078090; doi:10.18632/oncotarget.8909)

# SUPPLEMENTARY TABLES

**Supplementary Table S1: Structures of thalidomide analogs exhibiting anti-angiogenic activity in the Fli1:EGFP zebrafish model of angiogenesis**

C4
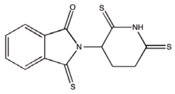

C11
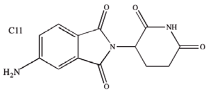

C14
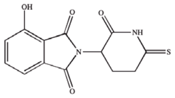

C17
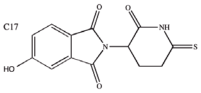

C19
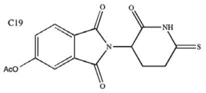

C34
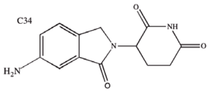

C51
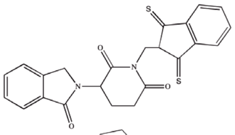

C55
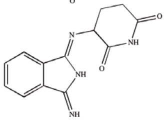

C60
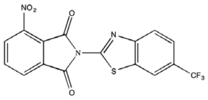

C69
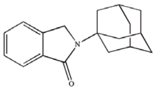

C70
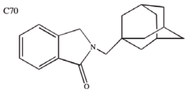

C73
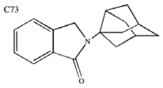

C81
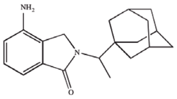

C83
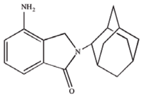

C84
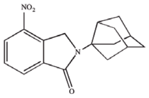

C89
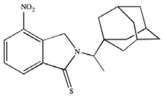

C91
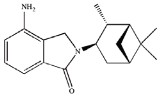

C93
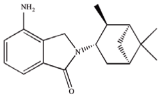

**Supplementary Table S2: Structures of thalidomide analogs which reduced neutrophil migration in the TG(MPO::EGFP)114.** zebrafish model of inflammation. Astericks indicates a teratogenic compound.

C2
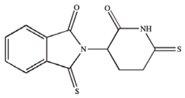
C59
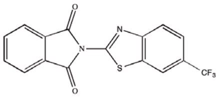
C5*
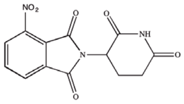
C7
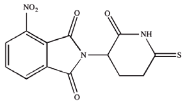
C64
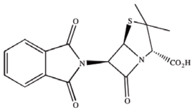
C24*
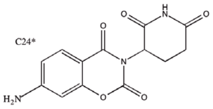
C9
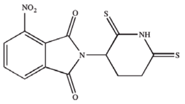
C65
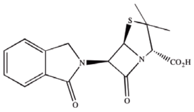
C29*
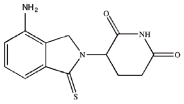
C46
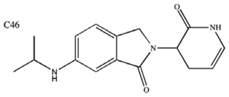
C72
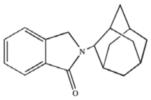
C32*
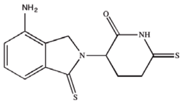
C53
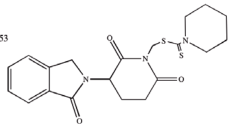
C74
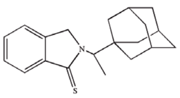
C33*
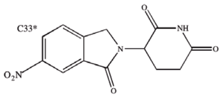
C56
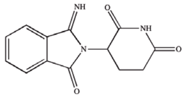
C77
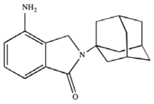
C67*
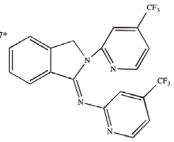
C58
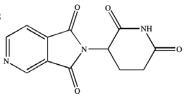
C86
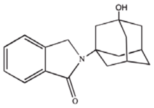
C76*
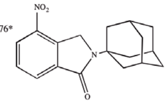

**Supplementary Table S3: Structures of thalidomide analogs showing both anti-angiogenic and anti-inflammatory activities in the Fli1:EGFP and TG(MPO::EGFP)114 zebrafish**

C18
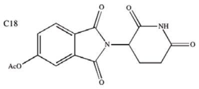
C52
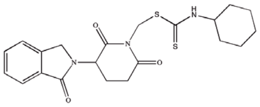
C66
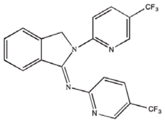
C20
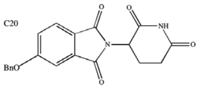
C61
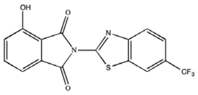
C68
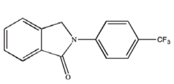
C23
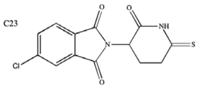
C62
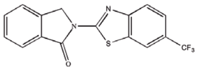
C82
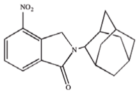
C30
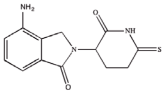
C63
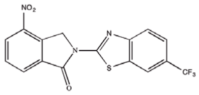
C85
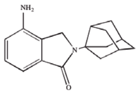
C57
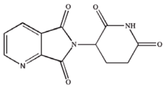

Supplement: Supplementary file 1 [file oncotarget-07-33237-s001.pdf]
